# Paton Prize Lecture Winner 2024: Gordon Holmes and the Irish spirit of adventure: Lessons for modern thinkers

**DOI:** 10.1113/EP092192

**Published:** 2025-01-28

**Authors:** Seán M. Roe

**Affiliations:** ^1^ Centre for Biomedical Sciences Education, School of Medicine, Dentistry and Biomedical Science Queen's University Belfast Belfast UK

**Keywords:** battle medicine, history of medicine, neuroanatomy, neurology, neurophysiology, neuroscience, war

## Abstract

This lecture is given in honour of Sir William Paton (1917–1993), physiologist, pharmacologist and Fellow of the Royal Society. His passion for the history of medicine led to generous donations to the Society, who consequently founded the Paton Prize Fund for historical research. After his death, this eponymous Prize Lecture was debuted in 1994. It has been a singular honour for me to acclaim the similarly influential Irish neurologist Gordon Morgan Holmes (1876–1965), whose work has been a particular preoccupation of mine for my entire career, since I first heard of him as an undergraduate in 1991. Holmes’ work on the World War I battlefield completely transformed neurology, in terms both of clinical practice and its knowledge base. Clinical techniques developed by him at that time remain established current practice, and his visual field maps were not superseded for 73 years. His legacy to neurology is extensive, with his editorship of the journal *Brain* from 1922 to 1937. He had a profound influence both here and across the Atlantic, evinced by global warm tributes published on his death in 1965. The Lecture concentrates on a confluence of events and circumstances existing in the Flanders trenches that met a man singularly suited to overcoming the challenges posed. He received training in neuroanatomy from the German anatomists and clinical skills from the British greats in the National Hospital Queen Square. Coupled with an Irish adventurousness and insatiable curiosity, this experience presaged advances that resonate >100 years on. His legacy speaks profoundly of curiosity, interdisciplinarity and reconciliation.

## Abstract (highlights)

- **What is the topic of the review?**
  This review concerns the life and career of the Irish neurologist and battle medic, Gordon Morgan Holmes (1876–1965). It focuses on how a unique set of circumstances combined with an equally unique skillset in Holmes to advance neurology over his 3 years on the World War I battlefield.
- **What advances does it highlight?**
  Clinical techniques developed by him at that time remain established current practice, and his visual field maps were not superseded for 73 years. His life emphasizes the importance of a multidisciplinary approach, embracing physiology, anatomy, clinical science and art, and illustrates the folly of restricting thought to simple subject areas.

## 1 SET‐UP

In the spring of 1991, as an undergraduate in University College Dublin's Earlsfort Terrace Campus, I was set a conundrum that has occupied me for my professional life. The late, great University College Dublin Scholar of Neurology and the History of Medicine, Professor Caoimhghin Breathneach (1923–2019) had been tasked with presenting histories of great figures in Irish Medicine in display cases outside the main lecture theatre. One of these concerned Sir Gordon Morgan Holmes, who during his life, had transformed the clinical practice of neurology alongside our knowledge of the visual cortex and the cerebellum. The question was thus set: ‘What was an Irishman, educated in Frankfurt by the German neuroanatomists doing on the Western Front in 1915 working for the British Expeditionary Force, and how did he achieve so much in three short years?’. This set the stage for a remarkable tale of confluence and coincidence, in which the right man with the right skills and attitudes alighted upon the exact circumstances to advance neurology by ∼70 years in three short, intense years in Flanders and northern France.

## 2 HOLMES, THE MAN (EARLY LIFE)

Born on 22 February 1876, Holmes spent his early life in Dellin House, Castlebellingham, a small townland 40 miles from Dublin (Breathneach, 1975). The early loss of his mother delayed the start of formal schooling, but he later detailed how he overcame this by teaching himself to read. It is also recalled that his first schoolmaster volunteered to give extra tuition, recognizing Holmes's extraordinary abilities, even at that stage (Walshe, 1966). Further early indications of what was to come lie in the history of his first school, the Dundalk Educational Institution, which produced three Victoria Cross awardees along with the most outstanding Irish fighter pilot of the First World War, George McIlroy (McCrea & Patterson, 2014). He went on to study Natural Science and Medicine in Trinity College Dublin, being senior moderator and gold medallist in 1898. His initial postgraduate adventures led to his serving as a ship's surgeon on board a vessel travelling to New Zealand. On his return to Ireland, he served as a resident medical officer in the Richmond Asylum (now St. Brendan's Psychiatric Hospital, Dublin). The earnings from these posts and the Stewart Scholarship in Nervous and Mental Diseases for Excellence in Anatomy from Trinity Medical School served to fund a trip to Europe to seek postgraduate education (Fine et al., 2011). These trips culminated with 2 years in the Senckenberg Institute in Frankfurt, where he encountered the first two major influencers of his career, Professors Ludwig Edinger and Carl Wiegert.

## 3 HOLMES THE INVESTIGATOR, GAINING HIS ECLECTIC SKILLSET

### 3.1 The German neuroanatomists

**FIGURE 1:**
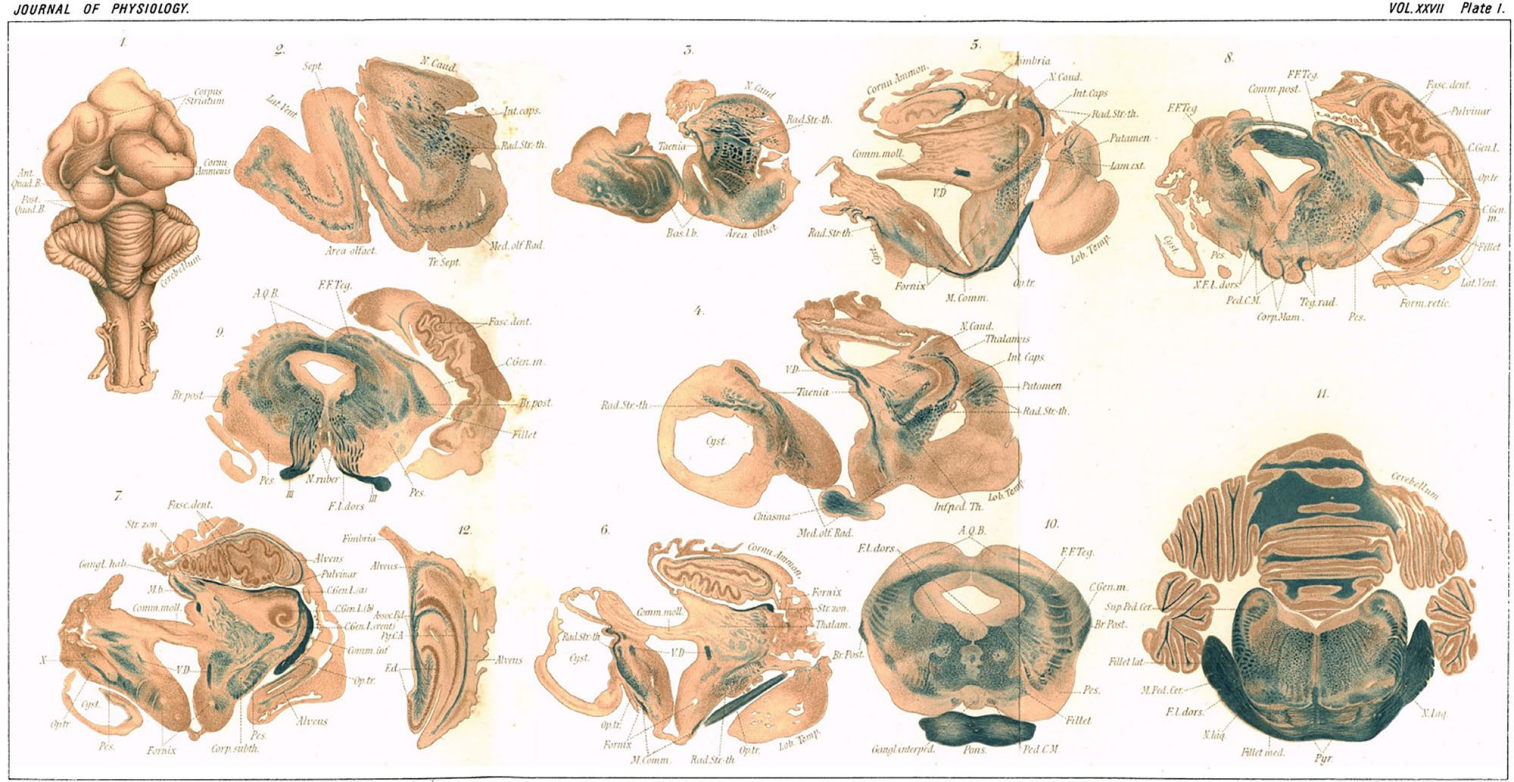
Holmes's artistic flair can be seen in this panel from his 1901 article in *The Journal of Physiology* on Goltz's ‘dog without a forebrain’ (Holmes, 1901).

Initially, Holmes's time in the Senckenberg Institute was taxing. In later conversations between his daughter Kathleen and Gerald Parsons‐Smith, she would detail how Herr Professor Edinger would tear up painstakingly drawn spinal cord sections that had taken her father 2 days to do (McDonald, 2007). Shortly afterwards, however, it was evident that the relationship warmed, with Edinger donating Holmes the preparation of Goltz's famous ‘dog without a forebrain’ for detailed histological study. The beautiful drawings resulting from this were published in *The Journal of Physiology* (Holmes, 1901) (Figure 1) and show an evident artistic flair in the young neuroanatomist. The skills of painstaking observation, mapping, documentation and drawing gained in Frankfurt would prove invaluable 15 years later, on the battlefields of Northern France and Flanders, in describing and mapping wounds to the visual cortices of his patients. Plans to extend his visit to the Senckenberg Institute were shelved when funds for his position were instead used to hire Paul Ehrlich (Fine et al., 2011). Consequently, Holmes went with letters of recommendation from Edinger to William Gowers and Victor Horsely and applied for a vacancy at the National Hospital, Queen Square, London (Coakley, 1992).

### 3.2 London, encountering a ‘who's who’ of physiology and neurology

Armed with Edinger's recommendation, Holmes initially approached William Gowers, calling at his home during dinner, being swiftly rebuffed after a peremptory interview in the hall. Unabashed, he visited Victor Horsely. Horsely had been enthusiastic about Holmes's work at the Senckenberg Institute and immediately arranged for his appointment as House Physician to John Hughlings Jackson at Queen Square (Coakley, 1992). It is said that in Holmes's tutelage under Jackson, the German and British neurology traditions came together. In Frankfurt, Edinger had emphasized the functional significance of anatomical investigations, and under Jackson the ‘unresting contemplation of facts of observation, scrupulously and untiringly acquired’ was emphasized (McDonald, 2007).

During his time at Queen Square, Holmes's collaborations and associations read like a list of the foremost practitioners of neurology and physiology. There is an account of weekend bowling trips with Henry Head, Victor Horsely and Ernest Henry Starling (Coakley, 1992). Notable research carried out at the time, with particular relevance to his subsequent work on the Western Front, were collaborations with Henry Head on sensory disturbances from cerebral lesions (Head & Holmes, 1911), a description of familial cerebellar degeneration in 1908 and, with Thomas Grainger Stewart, a characterization of the clinical signs of cerebellar dysfunction resulting from their observations of 40 patients with cerebellar tumours (Fine et al., 2011; Stewart & Holmes, 1904). The last of these, in particular, foreshadowed his work on cerebellar injury on the Western Front in 1917, reading like a thorough apprenticeship in making clinical determinations on cerebellar function.

A lucky escape came in 1910. With his desire for travel and adventure still unsatiated, Holmes discussed with Sir Robert Scott the possibility of joining his next expedition. Before Holmes could make any definitive plans, however, he ruptured his Achilles tendon and waved goodbye to the explorer from the quayside as he set off on his last ill‐fated expedition to the South Pole (Coakley, 1992). The desire for travel and adventure in Holmes would be thoroughly satisfied, however, 4 years later in Northern France and Flanders.

## 4 CONFLICT: THE OUTBREAK OF WAR AND THE IRISH CONTEXT

The outbreak of war in August 1914 coincided with the Home Rule crisis in Ireland, with the Nationalist Struggle for Independence, led by John Redmond, and the opposing Unionist faction of Edward Carson, who formed the Ulster Brigades in response. By September 1914, John Redmond had the Irish Home Rule bill on the Statute books, with Carson ready to oppose this violently with his Ulster Volunteer Force. On both sides, however, local hostilities were suspended with Redmond's famous September 1914 speech to journalists on the Golf Course of Woodenbridge County Wicklow [‘account for yourselves as men, not only for Ireland itself, but wherever the firing line extends’ (McGreevy, 2018)] and the incorporation of Carson's Ulster Volunteer Force into the 36th Ulster Division (Kennedy, 2005). It proved to be the greatest mobilization of Irish military manpower before or since.

**FIGURE 2:**
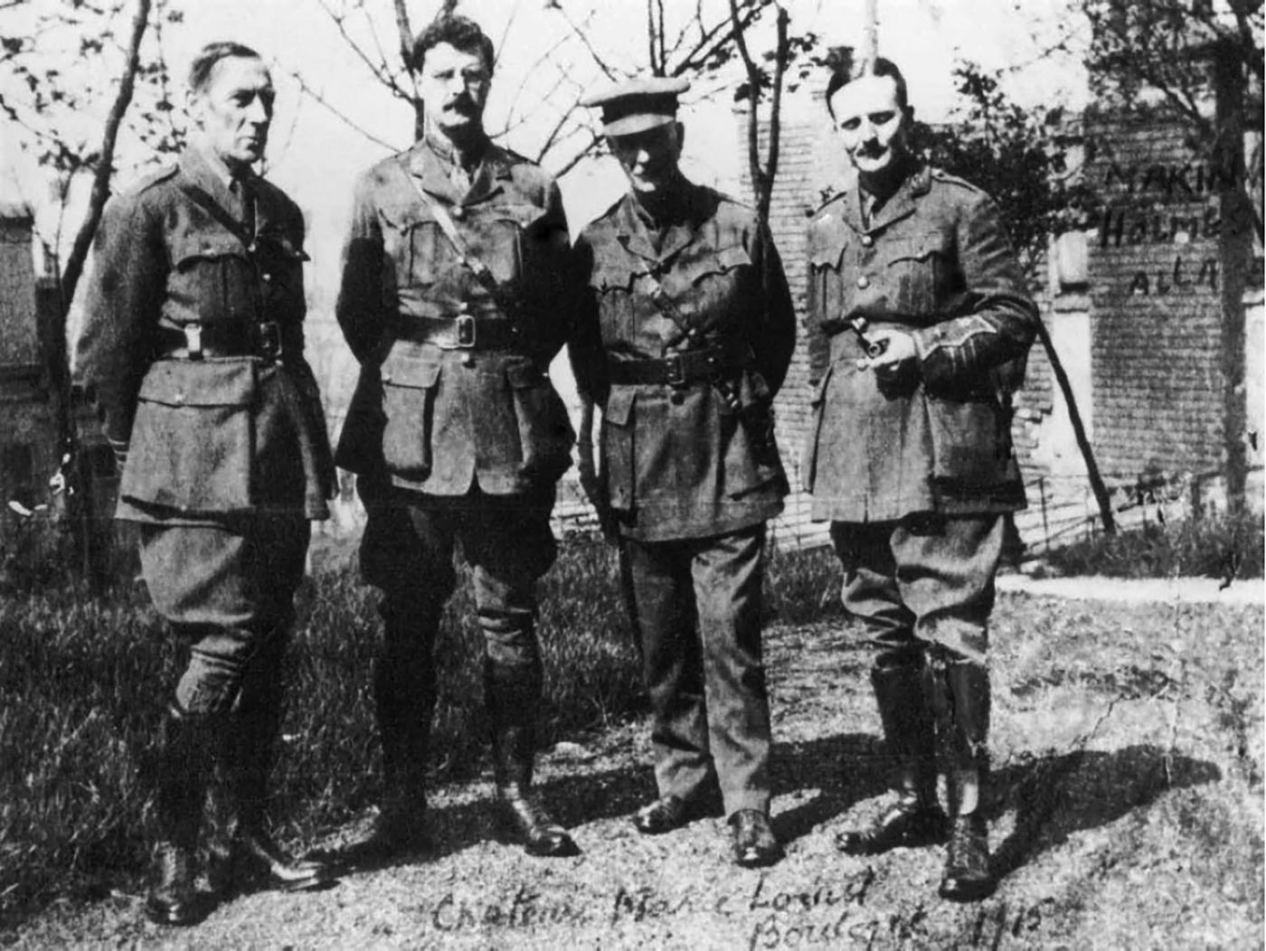
Gordon Morgan Holmes (second from left) in his British Expeditionary Force uniform on the Western Front in 1915. (Credit: Raven Press, Physiology News Licence CC BY‐SA 4.0.)

There was similar enthusiasm in the medical profession, with 5000 Irish doctors enlisting for service on the Western Front, forming the greatest Irish medical migration in history (Durnin, 2019). Holmes immediately applied for a commission in the Royal Army Medical Corps but was refused on the basis of myopia and migraine. Undeterred, he volunteered for the Red Cross with Percy Sergeant, another surgeon from Queen Square. Their success in a hospital immediately behind the front line led to the Royal Army Medical Corps reconsidering their decision and appointing Holmes as consultant neurologist to the British Armies in France, with the rank of Lieutenant Colonel (Figure 2).

## 5 THE WESTERN FRONT 1915–1918

### 5.1 Harvey Cushing's war diary

We are fortunate to have the account of Harvey Cushing, a contemporary of Holmes on the Western Front. Cushing was a professor of surgery at Harvard who had volunteered to liaise with the Royal Army Medical Corps in France, spending the month of April 1915 there, returning again to the Front as a US Army Major as soon as the USA entered the war 2 years later. His war diary ‘*From a Surgeons Journal, 1915–1918*’ (Cushing, 1936) gives great insight into conditions on the Western Front and the activities of many notable figures, including Holmes, Sergeant and William Osler. He was a particular admirer of the work of Holmes and Sergeant, noting both the conditions in which they worked (‘There are 900 acutely ill soldiers, convoys of 300 wounded might arrive in a day and there were only 10 doctors’) and the potential the conditions offered for research [‘with the proper backing, these two men (Holmes and Sergeant) have an unparallelled opportunity, not only to be of service to the individual wounded, but, when it is over to make a contribution to physiology, neurology and surgery which will be epochal’] (Fishman, 1997). In <9 months of war, Cushing mused, Holmes had amassed a lifetime's work (Lepore, 1994).

### 5.2 A confluence of coincidences

The Western Front was thus a stage for some of the most important scientific and clinical advances of the 20th century. A confluence of coincidences met a scientist uniquely disposed to take advantage of these circumstances.

**FIGURE 3:**
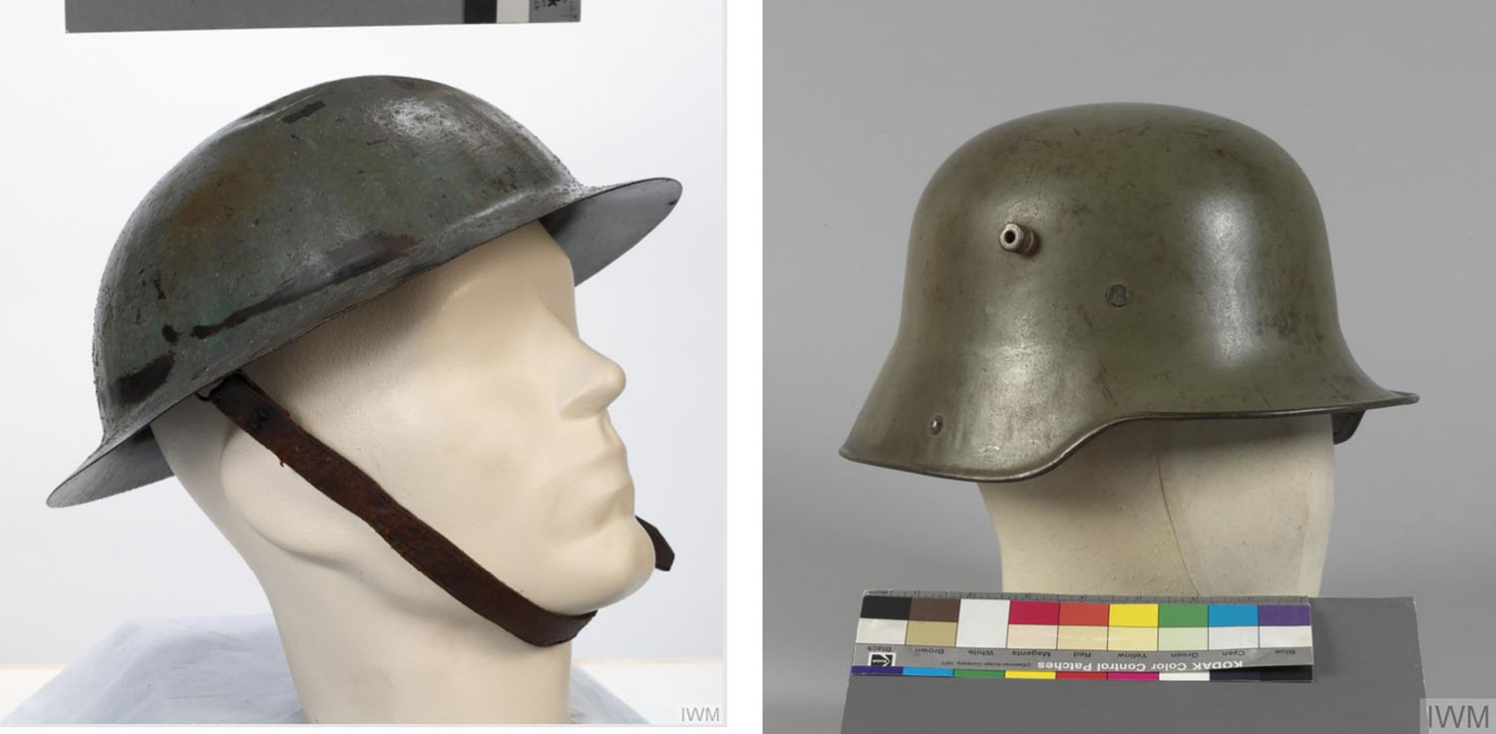
Comparison between the 1916 helmet designs used on the Western Front. The Brodie ‘Tommy’ helmet (left) sits high on the head and provides little protection from ground burst shrapnel. Contrast this with the German Stahlhelm ‘Coal Scuttle’ helmet (right), which provides much greater protection to the occipital lobe and cerebellar regions of the brain. (Images courtesy of the Imperial War Museum.)

Firstly, the Brodie Helmet used by the British Expeditionary Force on the Western Front was designed to be produced cheaply and easily from a single stamping of metal (Shadrake & Pughe, 2014). It was derived from the medieval British ‘Kettlehat’, whose design echoed the British preoccupation since Agincourt with archers and descending fire (Tribble, 2015). Thus, it provided good protection from airburst shells but not from shrapnel rounds that burst close to ground, projecting shell fragments into the exposed occipital lobe and cerebellum. As Cushing dryly observed at the time, ‘British Helmets danced on top of their heads, unlike those of the Germans’ (Figure 3, Cushing, 1936; Fishman, 1997)

Secondly, the weaponry used on the Western Front was unique in causing discrete and limited damage to the brain. For the first time [with the advent of the Mosin‐Nagant M‐1891 rifle first used in the Russo‐Japanese war of 1904, followed by the Mauser Gewehr 98 and the Short Magazine Lee‐Enfield (SMLE) of the First World War], rifles had sufficient power and accuracy to cause trauma at ranges seen in trench warfare (Lepore, 1994). The higher muzzle velocity of smaller, pointed projectiles allowed them to penetrate the skull (and helmet) while not achieving the lethal shock wave and cavitation effects of modern weapons. The Western Front also had battles often initiated with artillery barrages of shrapnel and high‐explosive shells; in the week preceding the Battle of the Somme, >1.5 million shells were fired at enemy trenches (Axelrod, 2016). Thus, soldiers often survived combat with damage localizable via entry and exit wounds and with focal neurological deficits that could be described by someone with the skills and motivation to do so. As Holmes himself noted in his seminal paper on cerebellar injury, ‘The opportunity of making uncomplicated clinical observations is rare in civil life, since acute lesions of the cerebellum, comparable with those produced by physiologists, are uncommon; tumours and abscesses which develop in it are very liable to compress or influence the functions of other parts too.… In warfare, on the other hand, wounds limited to the cerebellum and injuries of it of different extent and localization, can be frequently observed’ (Holmes, 1917).

Thirdly, Holmes's training in neuroanatomy, physiology and clinical observations equipped him uniquely to take advantage of these unique circumstances. His sense of adventure and dedication to duty kept him in the right place (under fire in casualty clearing stations), at the right time, in the exact circumstances that enabled the advances detailed here.

Finally, a central point is that, with the advances in battlefield medicine, wound care, sanitation and general hygiene, the First World War was perhaps the first time in history when a soldier's chance of being hit (and surviving) was vastly greater than his chance of becoming ill (Lepore, 1994), providing plenty of subjects for examination.

## 6 SEMINAL WARTIME PAPERS: THE VISUAL CORTEX

### 6.1 What was known in 1916

Prior to the early 20th century, little was known on the localization of visual function in the cerebral cortex, with studies in the 1870s and 1880s being limited to inflicting large, blinding lesions in the occipital and parietal cortices of dogs and monkeys (Fishman, 1997). This changed in the late 19th and early 20th centuries with the advent of rifles with sufficient accuracy and power to cause discrete and limited damage to the cortex without causing death. The relatively small cortical lesions could then be correlated with visual fields. In 1905, the Japanese and Russians went to war with the new rifles (the aforementioned Mosin‐Nagant M‐1891). A young Japanese doctor, Tatsuii Inouye, was assigned by his government to examine brain‐injured soldiers and their visual fields to determine the size of the pension due to them. Inouye developed a system of coordinates using external skull landmarks to relate to internal brain anatomy and, for the first time, accurately mapped central vision to the occipital pole. Although he published this finding in a German monograph in 1909 (Inouye, 1909), it went largely unnoticed except, notably, by Holmes, who cited it in his first work with Lister (Lister & Holmes, 1916). It is apparent that Holmes took Inouye's innovations and added his own experience in neuroanatomical mapping, observation and functional visual field testing to create something that has proved seminal to our understanding of visual representation in the occipital cortex to this day.

### 6.2 Holmes the neuroanatomist, pathologist, artist and clinician

**FIGURE 4:**
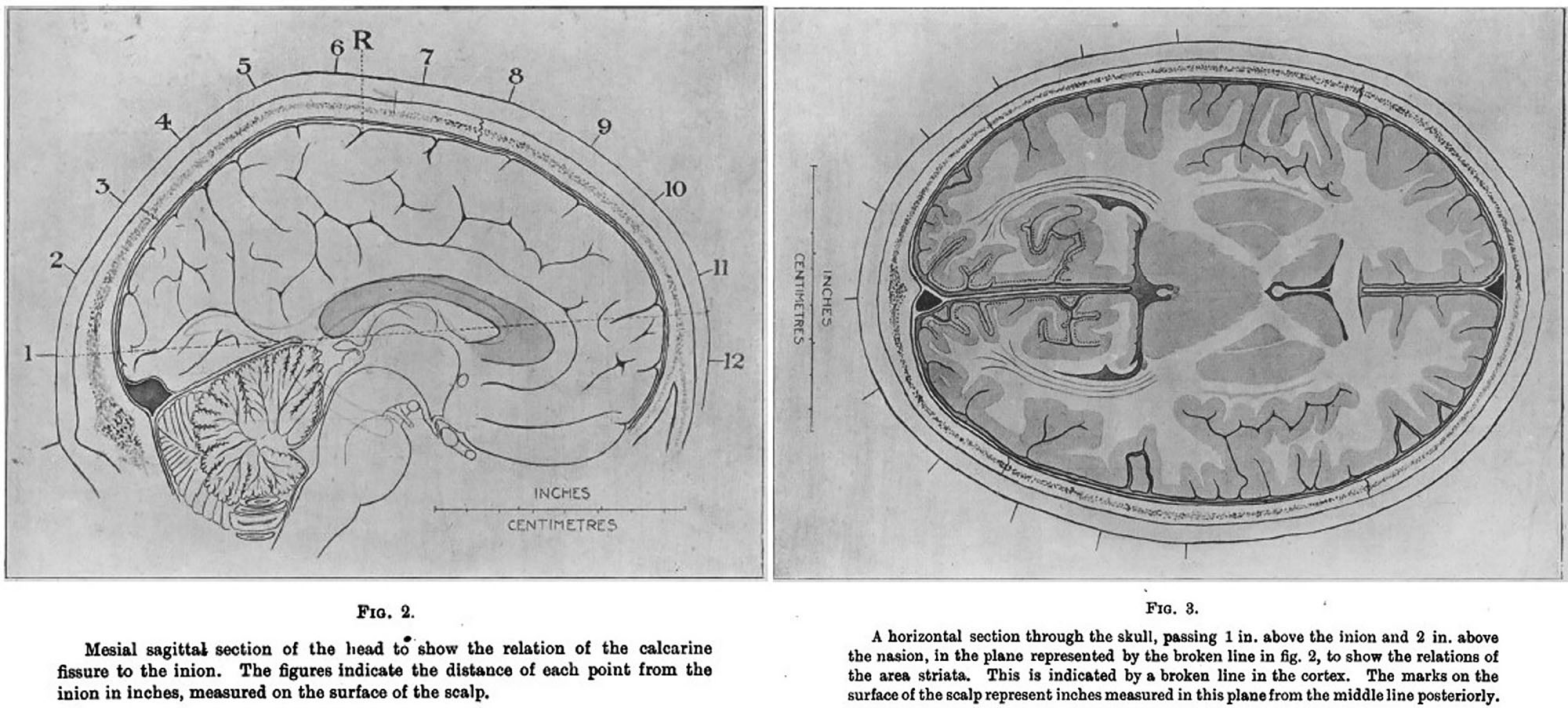
Simple mapping system developed by Holmes, whereby he could localize injuries to the visual areas by inferring the track of a projectile from entry and exit wounds, using the inion as a mapping reference (from Lister & Holmes, 1916).

**FIGURE 5:**
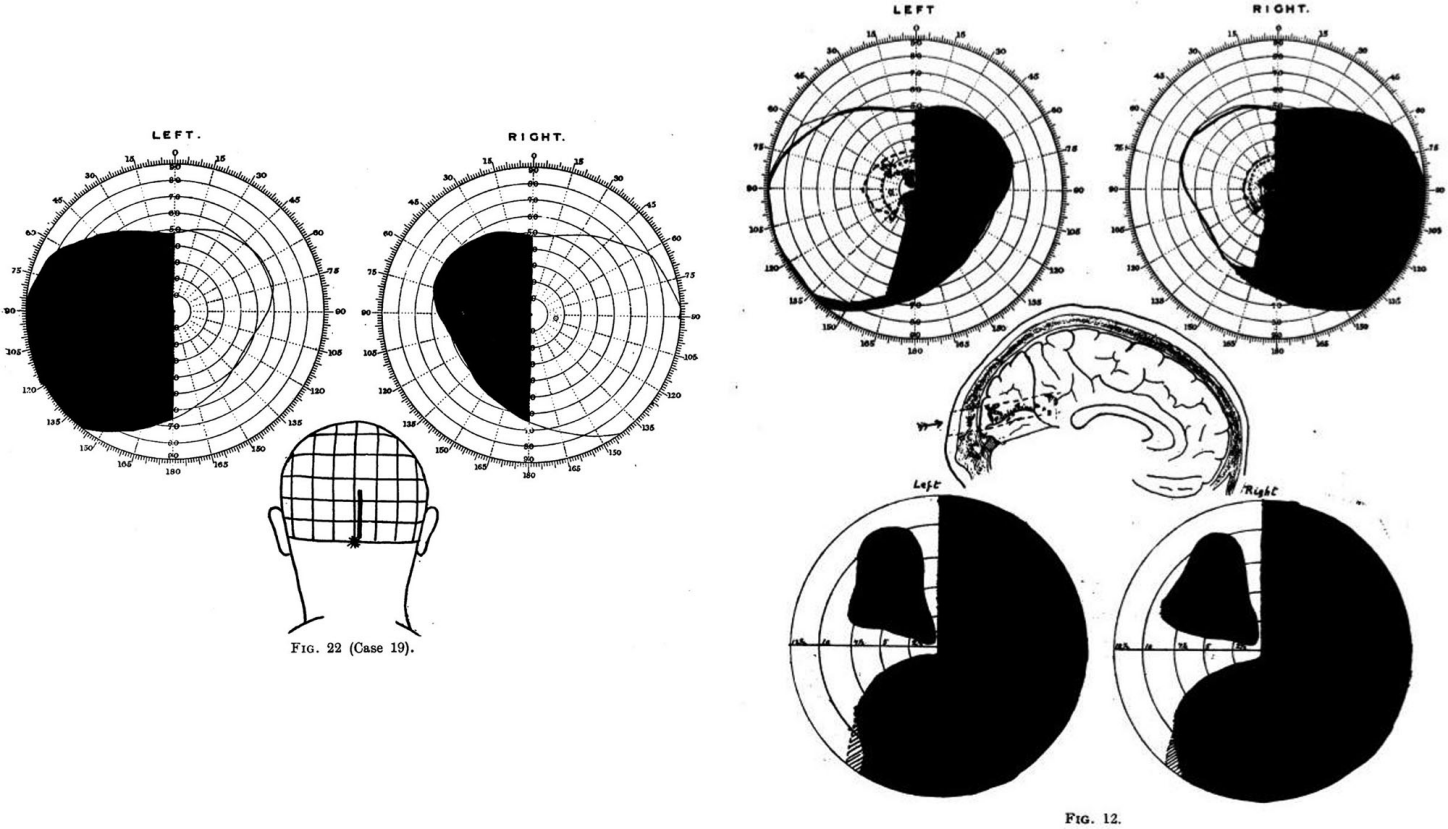
In the left panel is a visual field map of a patient featured in Lister & Holmes's 1916 paper with corresponding wound tract mapped out on a diagram of the skull as described in Figure 4. By 1918, Holmes had refined this technique to give a more accurate estimation of the passage of the projectile, in addition to incorporating an accurate foveal map (right panel, Holmes, 1918).

In his 1916 article (Lister & Holmes, 1916), Holmes describes in detail how he initially mapped entry and exit points of penetrating wounds of the skull using surface landmarks on the skin to relate them to brain structures (Figure 4). Also described were the various means by which he tested visual fields in soldiers. Different techniques were used depending on the infirmity of the subjects. Those confined to bed had their visual fields measured repeatedly using a hand perimeter, progressing to a McHardy perimeter when they were more mobile, with paracentral perimetry eventually performed with a large Bjerrum's screen. Contemporary accounts detail how Holmes travelled the cold frontline hospitals in his ‘British Warm’ (army‐issued greatcoat) to perform perimetry, only retiring to consolidate his case records late at night (Lepore, 1994). On the rare occasions when patients died of infection after visual field examination, Holmes performed the post‐mortem brain examination himself, correlating visual field deficits with observed brain pathology. Some of the resulting wound maps, with visual field representations are shown in Figure 5.

**FIGURE 6:**
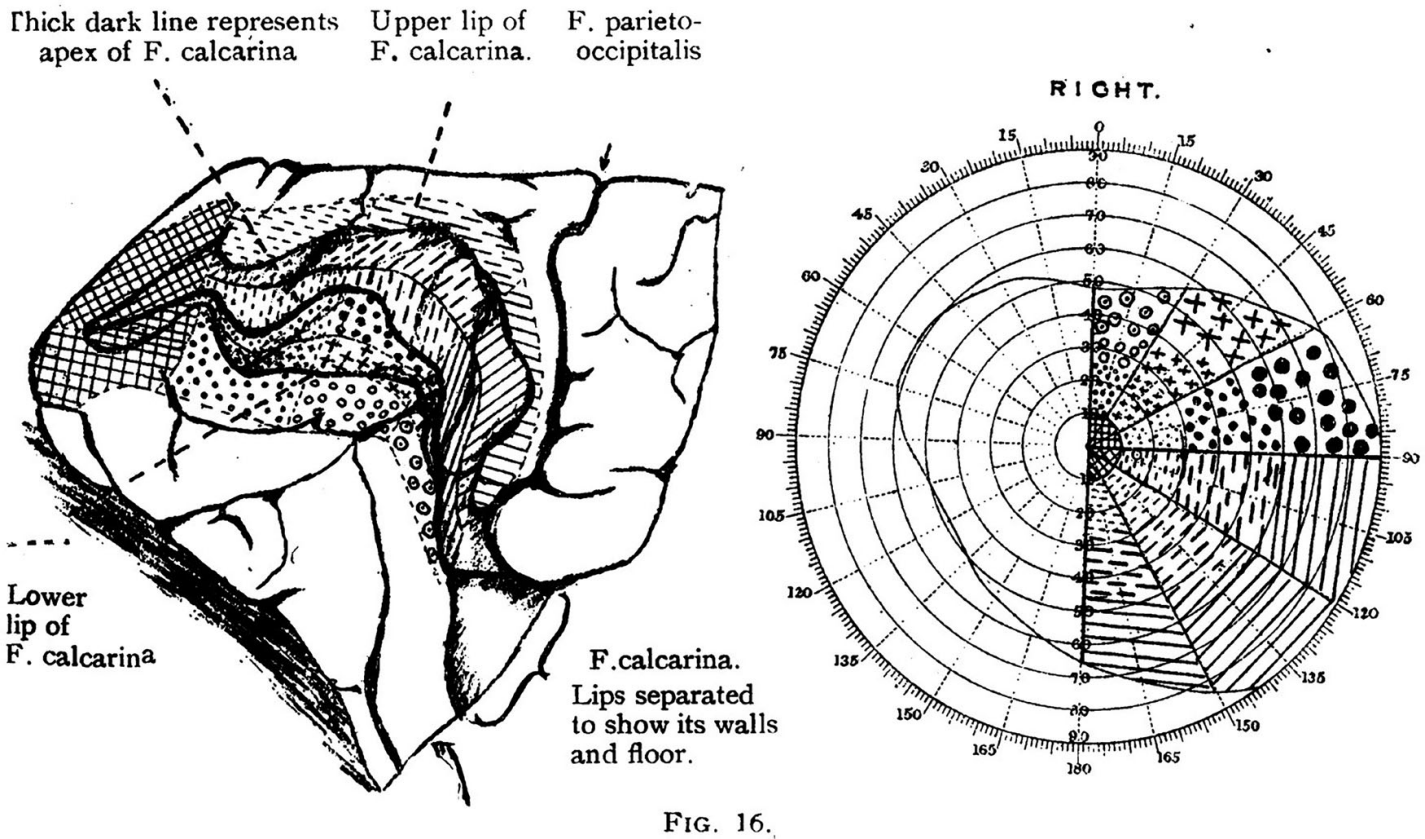
Holmes's map of the ‘cortical retina’, with corresponding visual field areas extrapolated. Between the work by Lister and Holmes (1916) and his 1918 paper, 35 cases were presented, but 400 cases were used during the conflict to establish the technique (Holmes, 1918).

Techniques developed in the 1916 paper were refined in 1918 (Holmes, 1918; Lister & Holmes, 1916), and although a total of 35 cases were presented over the two papers, >400 cases were studied in the development and refinement of the techniques. The resulting visual field maps became known as the ‘classical Holmes maps’ (Figure 6). They were widely reprinted and became the standard illustration of the cortical retina. They persisted for 73 years, surviving two World War II studies of head wounds (Fishman, 1997).

The classical Holmes maps were superseded only with the advent of sophisticated MRI techniques (Horton & Hoyt, 1991; Lindegger & Pless, 2019). Modern visual field maps ascribe 70% of the surface area of the striate cortex to the central 15° of visual field, whereas Holmes attributed only 25% to the central 15° portion. Holmes also established the orthodoxy (now understood to be incorrect) that the entire visual centre was located in V1, the area around the calcarine fissure that Holmes mapped. He did not allow for the parallel processing theories of Riddoch, who posited that different aspects of vision were coded in different areas of the occipital cortex, nor did he allow for the phenomenon of cerebral achromatopsia, in which a heminaopic field lacked colour while other aspects of vision (shape and movement) remained intact (Lepore, 1994). Holme's prestige was such that, after the War, mention of cerebral achromatopsia virtually disappeared from the literature. It was revived only in the 1970s and 1980s by neuroscientists (including the 2021 Paton Prize Lecture winner, Semir Zeki) who noted that, in monkeys, one could record from cortical cells that responded only to colour stimuli (Zeki, 2022). Holmes may be forgiven for this misapprehension; the brain area in the human corresponding to Zeki's V4 area serving colour processing was deep in the lingual and fusiform gyri. Penetrating wounds here would usually have been fatal, as Holmes himself (Lister & Holmes, 1916) admitted, giving him little opportunity to study such phenomena (Lepore, 1994).

## 7 SEMINAL WARTIME PAPERS: THE CEREBELLUM

### 7.1 The ‘Paganini of clinical practice’

A key to understanding Holmes's remarkable wartime work on the clinical features of cerebellar injury is the consideration of three factors. Firstly, Holmes had already worked extensively on cerebellar injury in his 1904 collaboration with Thomas Grainger Stewart. Here, 40 cases of cerebellar tumours were examined for their clinical manifestations, with extensive documentation of the attendant motor symptoms (Stewart & Holmes, 1904). Holmes's later wartime article on the cerebellum (Holmes, 1917) almost reads like a companion piece to the 1904 paper; it is evident that his earlier work served as an excellent apprenticeship. In Queen Square, under Stewart's tutelage, he developed the clinical and physiological techniques he would go on to use in field hospitals 12 years later. Secondly, Holmes relished the unique opportunity that circumstances on the Western Front offered to advance the field, evidenced by his quote on the advantages of ‘acute lesions of the cerebellum’ over those produced by tumours and abscesses in civil life (Holmes, 1917). He spoke later, in his 1944 Ferrier Lecture, on the competing challenges of the clinician and physiologist thus: ‘the physiologist is like the builder in ashlar or hewn stones which can easily be fitted together, while the physician resembles the mason who has to use irregular rubble and therefore requires more time and labour to attain his end’ (McDonald, 2007). Holmes evidently took full advantage of circumstances in which war brought the hewn materials of the physiologist and the physician's rubble closer together. Thirdly, Holmes's skill in eliciting clinical signs from patients was unparallelled at the time. In a contemporary account from Macdonald Critchley: ‘he could coax physical signs out of a patient like a Paganini on the violin … it was sheer delight to watch him evoking one physical sign after another in a patient with, say tabes … like some violent conjuror, he would demonstrate the typical zones of hypalgesia.… Every neurologist alive today‐wherever he works is unconsciously utilising the routine clinical examination propagated, perfected, and perpetuated by Gordon Holmes’ (Fishman, 1997). More humorous accounts of his wartime interactions with patients are given in Cushing's famous surgeon's journal, where he details an interaction between Holmes and a cockney soldier: ‘Y mean that bleedin’ big Irishman! E pulls this iron bar out of ’is arse and ’its me leg with it’ (Cushing, 1936).

**FIGURE 7:**
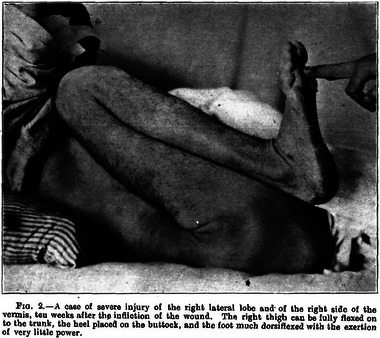
A picture from Holmes's work on cerebellar injuries showing the hypotonia resulting from severe injury to the right lateral cerebellum and vermis (Holmes, 1917).

**FIGURE 8:**
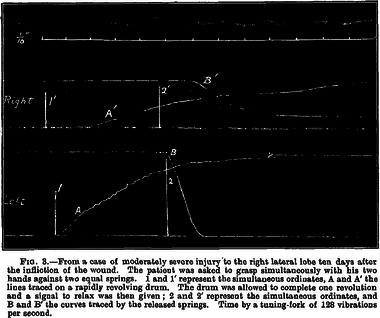
Smoke drum trace from Holmes's paper on cerebellar injuries showing what Holmes termed ‘Asthenia and Slowness in Muscle Contractions and Relaxations’. The contrast in the latency and strength of muscle contractions between the affected and non‐affected sides is evident here (Holmes, 1917).

**FIGURE 9:**
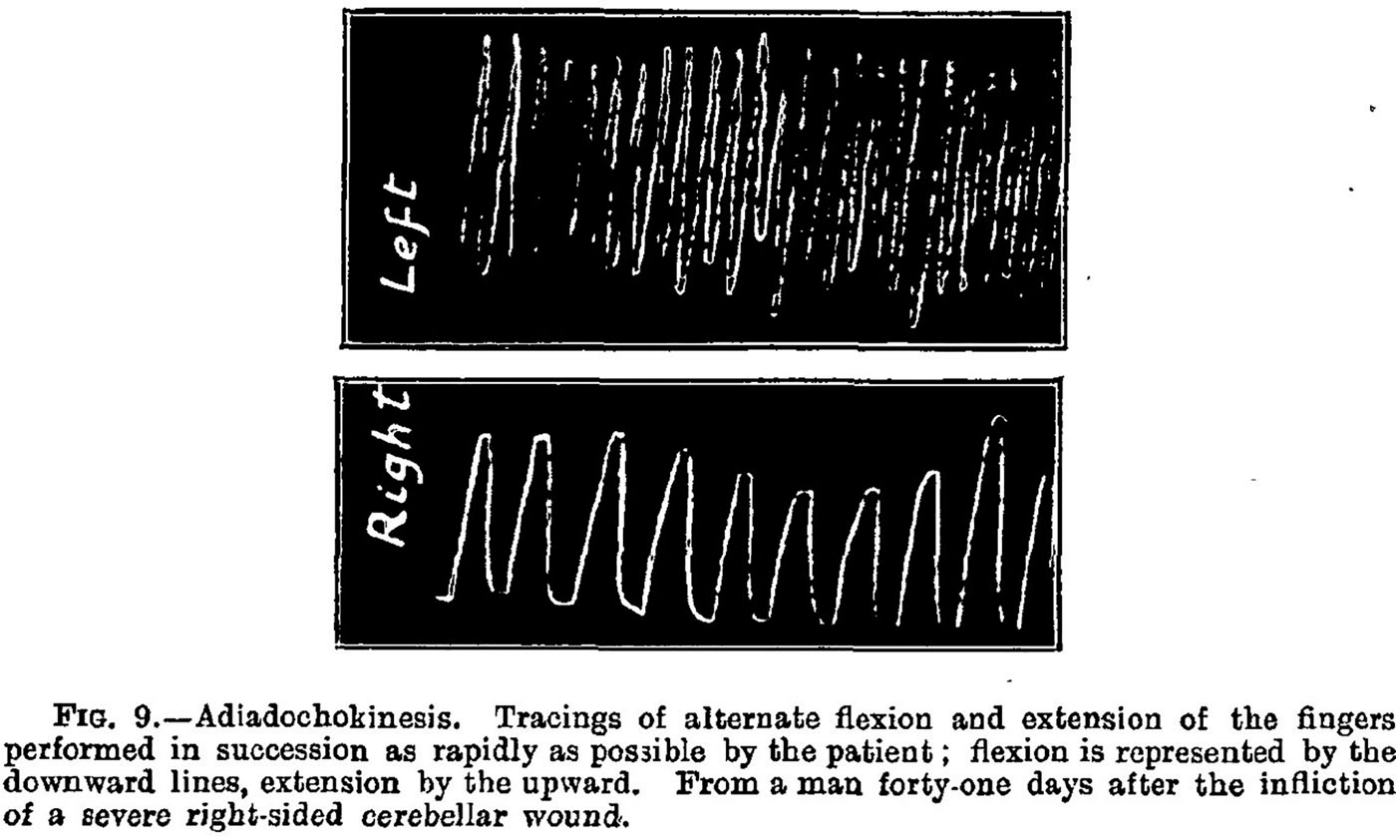
Smoke drum trace from Holmes's paper on cerebellar injuries showing the dysdiadochokinesia first described by Joseph Babinski in 1907 (termed ‘adiadochokinesis’ here) (Holmes, 1917).

The results from this coincidence of circumstances and unique skill are apparent in Holmes's comprehensive 1917 work on cerebellar injuries attributable to gunshots (Holmes, 1917). In compiling this treatise, Holmes used careful clinical observations (Figure 7) and the recording of pathologies of motor control using a primitive smoke drum apparatus (Figures 8 and 9). One can only imagine the difficulties of obtaining such precisely metered and timed traces on primitive equipment in rudimentary field hospitals. In this paper alone, Holmes established the basic clinical signs of cerebellar dysfunction, describing in detail asthenia, ataxia, movement decomposition, dysmetria, tremor, dysdiadochokinesia, handwriting effects, past pointing, reflex and gait disorders, ocular movement derangements and speech difficulties. A neurological protocol for testing motor control was established here, and a phenomenon (in which a patient tries to move a limb against a resistance, and when the resistance is removed the limb fails to move back to its original position after springing forwards) was named for Holmes. It was named the ‘Holmes rebound phenomenon’, although, more accurately, it should be referred to as the ‘lack of rebound’ phenomenon.

## 8 HOLMES'S LEGACIES

### 8.1 To the subject: Holmes's scientific legacy

Between 1915 and 1918 alone, Holmes produced 18 papers, the findings of which were summarized in four seminal lectures: the Goulstonian Lecture (on spinal injuries) in 1915, The Trinity College Dubin Montgomery Lecture in 1919 and the Ferrier Lecture in 1944 (both on the visual cortex), and the Croonian Lecture (on cerebellar injury) to the Royal College of Physicians in 1922. Although there were fundamental errors in his interpretation of visual processing, his maps of the cortical retina established under fire in northern France were not superseded until the advent of modern scanning technology (Horton & Hoyt, 1991). Between 1919 and 1956, he produced 102 publications and established a protocol of neurological testing that still remains. His influence on the subject as editor of *Brain* between 1922 and 1937 is undisputed.

### 8.2 As a mentee and a mentor

Among the most abiding lessons of Holmes's life is to pay attention to those around you, be they people from whom you may learn or to whom you may provide direction. As a young neuroanatomist, Holmes learned from Herr Professors Edinger and Wiegert. At Queen Square, he was exposed to the clinical expertise of Hughlings Jackson, Gowers, Sergeant, Lister and Horsely, in addition to the scientific rigour of Henry Head, Stewart and Starling. He, in his turn, profoundly influenced British neurology in teaching F. M. R. Walshe and Macdonald Critchley and changed the subject further afield in mentoring Denny‐Browne and Penfield. Penfield (who studied under other notables, such as Ramon y Cajal and C. S. Sherrington), stated in his warm obituary for Holmes that his old mentor was ‘one of the finest teachers he had known; beneath the exterior of a martinet, there was an Irish heart of gold’ (Penfield, 1967).

### 8.3 Reconciliation

The choice of an Irish neurology hero, working for the British Expeditionary Force during the First World War, is apposite to our current political context. Since the Good Friday Agreement of April 1998, the need for a shared Irish history embracing both Nationalist and Unionist traditions has become increasingly apparent. This has been evident on an Irish governmental level at least since April 1995, when the Irish World War I volunteers were first honoured by the Taoiseach, John Bruton (Department of the Taoiseach, 1995). In this 80th D‐Day anniversary year (2024), there has been much interest in Irish contributors to the allied cause, such as Brendan ‘Spitfire Paddy’ Finucane, Rickard Donovan, the Wexford Sailor who oversaw Operation Neptune (Naval aspect of D‐Day) and Maureen Sweeney, the County Mayo Meteorologist who caused Eisenhower to delay the invasion. All of these are signs of a nation taking the imprecations to embrace a shared history of both traditions to heart.

### 8.4 Interdisciplinarity

Holmes's legacy has relevance for modern interdisciplinarity. Across his work (and often in the same article) he might work variously as an anatomist, physiologist, pathologist, neurologist, clinician or visual artist. This eclectic attitude to adventure, curiosity and progress is probably best captured by J. Robert Oppenheimer from his 1953 Reith Lecture: ‘We read, we study, we recognise and love that union of the generally incompatible’ (Oppenheimer, 1955).

## AUTHOR CONTRIBUTIONS

Sole author.

## CONFLICT OF INTEREST

None declared.

## FUNDING INFORMATION

None.
